# Mangiferin and Taurine Ameliorate MSRV Infection by Suppressing NF-κB Signaling

**DOI:** 10.1128/spectrum.05146-22

**Published:** 2023-05-31

**Authors:** Caijiao Dai, Li Yu, Zhiwen Wang, Peng Deng, Lijuan Li, Zemao Gu, Xugang He, Jianghua Wang, Junfa Yuan

**Affiliations:** a Department of Aquatic Animal Medicine, College of Fisheries, Huazhong Agricultural University, Wuhan, People’s Republic of China; b National Aquatic Animal Diseases Para-reference laboratory (HZAU), Wuhan, People's Republic of China; c Bureau of Agriculture and Rural Affairs of Xianyou County, Putian, People's Republic of China; d Wuhan Academy of Agricultural Sciences, Wuhan, People’s Republic of China; e Hubei Engineering Research Center for Aquatic Animal Diseases Control and Prevention, Wuhan, People’s Republic of China; f Engineering Research Center of Green development for Conventional Aquatic Biological Industry in the Yangtze River Economic Belt, Ministry of Education, Wuhan, People’s Republic of China; Regional Centre for Biotechnology

**Keywords:** *Micropterus salmoides*, inflammation, oxidative stress, mangiferin, taurine, NF-κB signaling

## Abstract

The emergence or reemergence of viruses pose a substantial threat and challenge to the world population, livestock, and wildlife. However, the landscape of antiviral agents either for human or animal viral diseases is still underdeveloped. The far tougher actuality is the case that there are no approved antiviral drugs in the aquaculture industry, although there are diverse viral pathogens. In this study, using a novel epithelial cell line derived from the brain of Micropterus salmoides (MSBr), inflammation and oxidative stress were found to implicate the major pathophysiology of *M. salmoides* rhabdovirus (MSRV) through transcriptome analysis and biochemical tests. Elevated levels of proinflammatory cytokines (interleukin-1β [IL-1β], IL-6, IL-8, tumor necrosis factor alpha [TNF-α], and gamma interferon [IFN-γ]) and accumulated contents of reactive oxygen species (ROS) as well as biomarkers of oxidative damage (protein carbonyl and 8-OHdG) were observed after MSRV infection in the MSBr cells. Mangiferin or taurine dampened MSRV-induced inflammation and rescued the oxidative stress and, thus, inhibited the replication of MSRV in the MSBr cells with 50% effective concentration (EC_50_) values of 6.77 μg/mL and 8.02 μg/mL, respectively. Further, mangiferin or taurine hampered the activation of NF-κB1 and the NF-κB1 promoter as well as the increase of phosphorylated NF-κB (p65) protein level induced by MSRV infection, indicating their antiviral mechanism by suppressing NF-κB signaling. These findings exemplify a practice approach, aiming to dampen and redirect inflammatory responses, to develop broad-spectrum antivirals.

**IMPORTANCE** Aquaculture now provides almost half of all fish for human food in 2021 and plays a significant role in eliminating hunger, promoting health, and reducing poverty. There are diverse viral pathogens that decrease production in aquaculture. We developed a novel epithelial cell line derived from the brain of *Micropterus salmoides*, which can be used for virus isolation, gene expressing, and drug screening. In this study, we focus on *M. salmoides* rhabdovirus (MSRV) and revealed its pathophysiology of inflammation and oxidative stress. Aiming to dampen and redirect inflammatory responses, mangiferin or taurine exhibited their antiviral capability by suppressing NF-κB signaling. Our findings exemplify a practice approach to develop broad-spectrum antivirals by dampening and redirecting inflammatory responses.

## INTRODUCTION

The emergence or reemergence of viruses, such as severe acute respiratory syndrome coronavirus 2 (SARS-CoV-2), monkeypox (MPXV), influenza A virus (IAV), and African swine fever virus (ASF) (1–4), pose a substantial threat and challenge to the world population, livestock, and wildlife. In addition, pathogenic viral factors are responsible for high mortality rates in both freshwater and marine organisms, causing severe economic losses and ecological influence (5–6). However, the landscape of antiviral agents either for human or animal viral diseases is still underdeveloped. For instance, there are only 179 approved antiviral drugs, representing 4.4% of 4,051 approved medicines for humans (7). The far tougher actuality is that there are no approved antiviral drugs in the aquaculture industry. Virus-specific drug development and broad-spectrum antivirals (BSAs) approaches represent different strategies with relative merits (8, 9). Given the feature of BSAs that can inhibit the replication of multiple viruses, BSAs are considered to foster proper preparedness for newly emerged viruses (10–12).

Inflammation is a normal part of the body’s defense to injury or infection (13). However, it is detrimental to host fitness when inflammation occurs in healthy tissues or lasts too long. For instance, excess levels of proinflammatory molecules, known as cytokine storm, is a major cause of morbidity and mortality in many viral infections, including SARS-CoV-2 and IAV (14). Immunomodulatory therapeutic drugs aiming to dampen and redirect inflammatory responses show promise to exert the antiviral effects ideally. Due to its ability to promote the expression of numerous proteins involved in innate and adaptive immunity, nuclear factor-κB (NF-κB) may coordinate various aspects of immune function required for resistance to infection (15). Furthermore, activation of NF-κB is a hallmark of most viral infections, providing a potential target for development of BSAs.

Aquaculture now provides almost half of all fish for human food in 2021 and plays a significant role in eliminating hunger, promoting health, and reducing poverty (16). There are diverse viral pathogens that decreased production in aquaculture (17). As exemplified by Micropterus salmoides, farmed *M. salmoides* annually provided more than 62.1 thousand tons worldwide (18). Both wild and civilized *M. salmoides* have suffered from different diseases elicited by bacteria, parasites, and viruses in recent years, particularly viral diseases (19). Among these viral pathogens, *M. salmoides* rhabdovirus (MSRV) is of particular concern. MSRV-infected fish exhibit crooked bodies, corkscrews, and irregular swimming (20). There is an urgent need for antivirals, particularly BSAs, to cope with MSRV and diverse viral pathogens in the aquaculture industry.

In this study, a novel cell line, *Micropterus salmoides* brain cell (MSBr), was established from the brain of *M. salmoides* and was then used to investigate the pathological mechanisms of MSRV through RNA sequencing and biochemical tests. By dampening inflammatory responses and rescuing oxidative stress, mangiferin or taurine exhibited their antiviral capability by suppressing NF-κB signaling. This study presents a promising approach to develop BSAs by targeting NF-κB signaling.

## RESULTS

### Establishment and characterization of *M. salmoides* brain cells.

**FIG 1:**
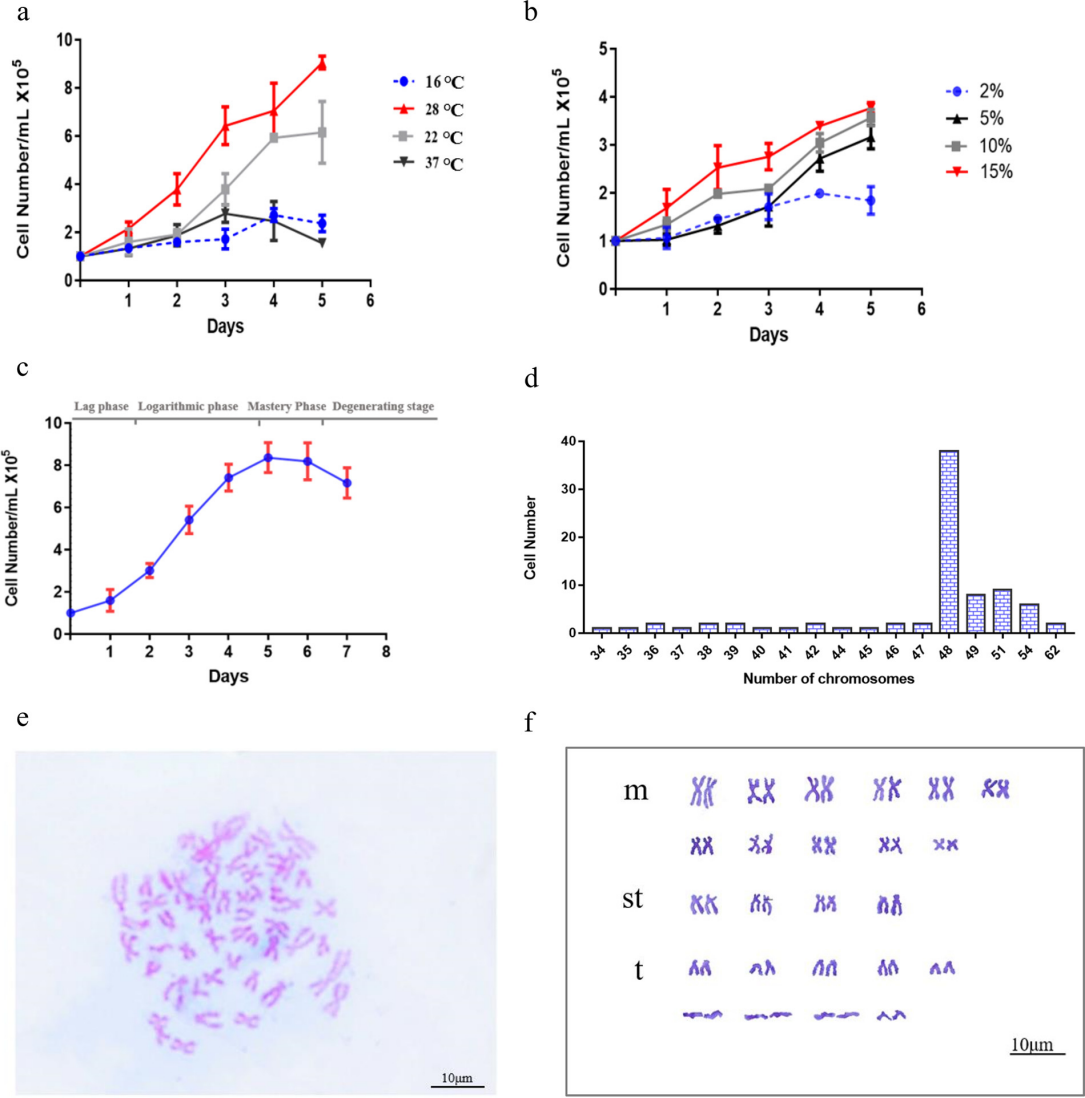
Biological characteristics of *Micropterus salmoides* brain cells and their chromosomal features. (a) Temperature dependence of *M. salmoides* brain cell growth. (b) Serum dependence of *M. salmoides* brain cell growth. (c) The growth curve of *M. salmoides* brain cells. (d) Distribution of chromosome numbers in 80 *M. salmoides* brain cells at passage 26. Cells were harvested by trypsinization and counted once per day. Three wells were included for each cell count. Bar, mean ± SD. (e) Karyotype of *M. salmoides* brain cells. (f) Main chromosome number of MSBr was 48, which consisted of 11 pairs of mediocentrics (m), 4 pairs of subtelocentrics (st), and 9 pairs of telocentrics (t) (2n = 22 m + 8 st + 18 t). Scale bar of images, 10 μm.

A novel cell line, *Micropterus salmoides* brain cell (MSBr), was derived from *M. salmoides* and has been subcultured >60 times over 9 months. MSBr cells consist primarily of epithelial-like cells and had the ability to express foreign genes efficiently (see Fig. S1 and S2 in the supplemental material). MSBr cells were able to grow at temperatures between 16 and 37°C with an optimum temperature of 28°C (Fig. 1a). The growth rate of MSBr cells increased when the proportion of fetal bovine serum (FBS) increased from 2% to 15% at 28°C (Fig. 1b). According to the growth curves of MSBr cells, the population doubling time was 49.06 h when cells grew in 10% FBS at 28°C (Fig. 1c). Chromosomal analysis revealed that the chromosome numbers of 80 metaphase MSBr cells varied from 34 to 62, with a median number of 48 at passage 26. As shown in Fig. 1d, approximately 47.5% of the cells had a diploid chromosome number of 2n = 48, and karyotype analysis indicated that MSBr consisted of 11 pairs of mediocentrics, 4 pairs of subtelocentrics, and 9 pairs of telocentrics (2n = 22 m + 8 st + 18 t) (Fig. 1e and f). Cytochrome *b* sequencing showed 99.47% sequence similarity between *M. salmoides* tissue and MSBr cells and supported their *M. salmoides* origin (data not shown).

**FIG 2:**
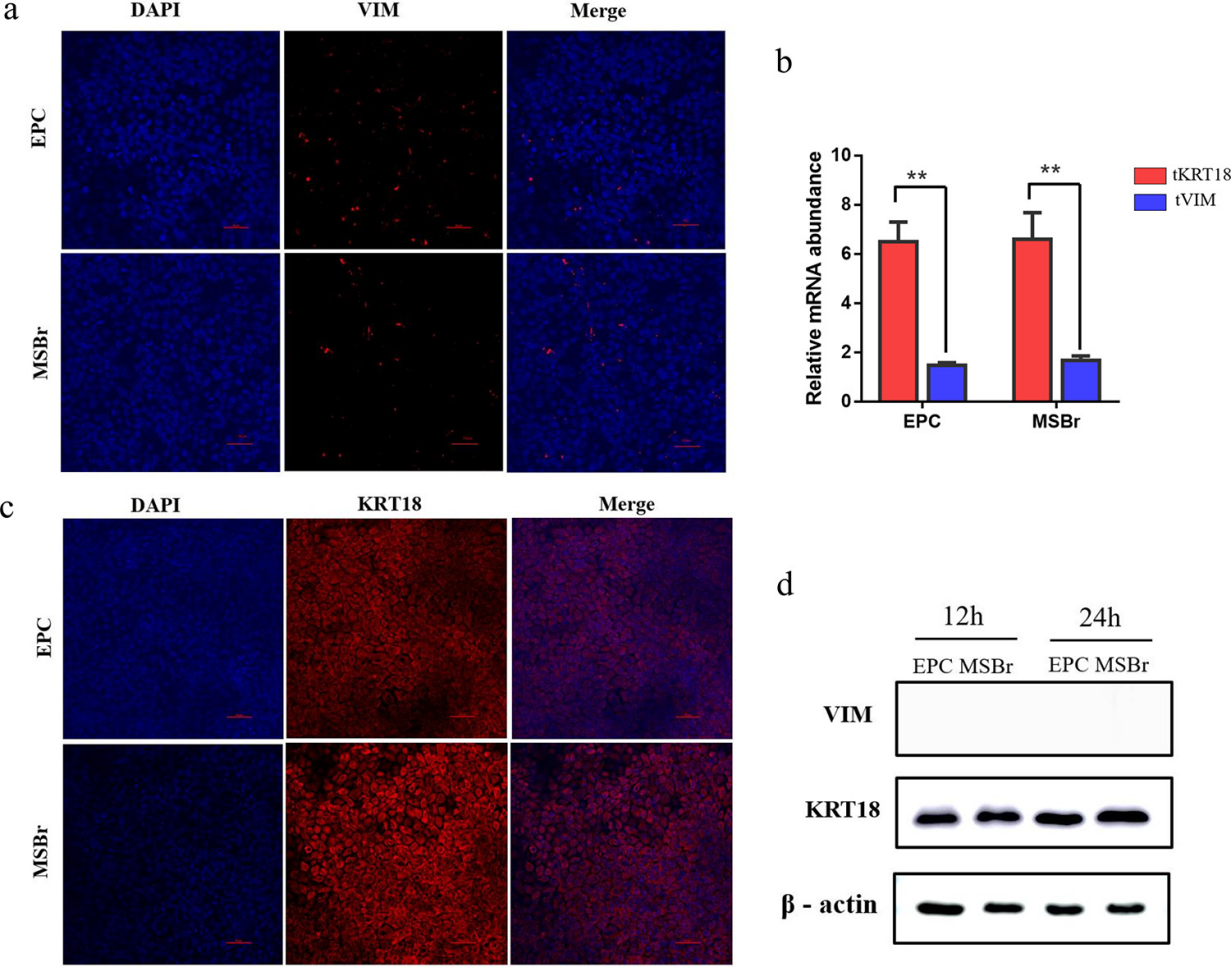
Expression of vimentin (VIM) and keratin 18 (KRT18) in the *Micropterus salmoides* brain cell line (MSBr) and epithelioma papulosum cyprini (EPC). (a) EPC and MSBr were stained with VIM antibodies (red), and cell nuclei were stained by DAPI (blue). Scale bar of images, 100 μm. (b) mRNA expression level of VIM and KRT18 were analyzed by quantitative RT-PCR. (c) EPC and MSBr were stained with KRT18 antibodies (red), and cell nuclei were stained by DAPI (blue). Scale bar of images, 100 μm. (d) The protein expression levels of VIM and KRT18 were analyzed by Western blotting assays at 12 h and 24 h after cells seed to the cell plate, respectively.

KRT18 and VIM were used as the markers to distinguish an epithelial or fibroblast cell. To determine the lineage of MSBr cells, the expressions of KRT18 and VIM were detected. Immunofluorescence analysis showed that MSBr cells were strongly positive for KRT18. No fluorescence signal was observed when MSBr cells were incubated with anti-vimentin antibodies (Fig. 2a and c). These results were further confirmed with the mRNA and protein levels of VIM and KRT18, indicating the epithelial origin of MSBr cells (Fig. 2b and d).

### Multiple fish viruses replicated efficiently in *M. salmoides* brain cells.

**FIG 3:**
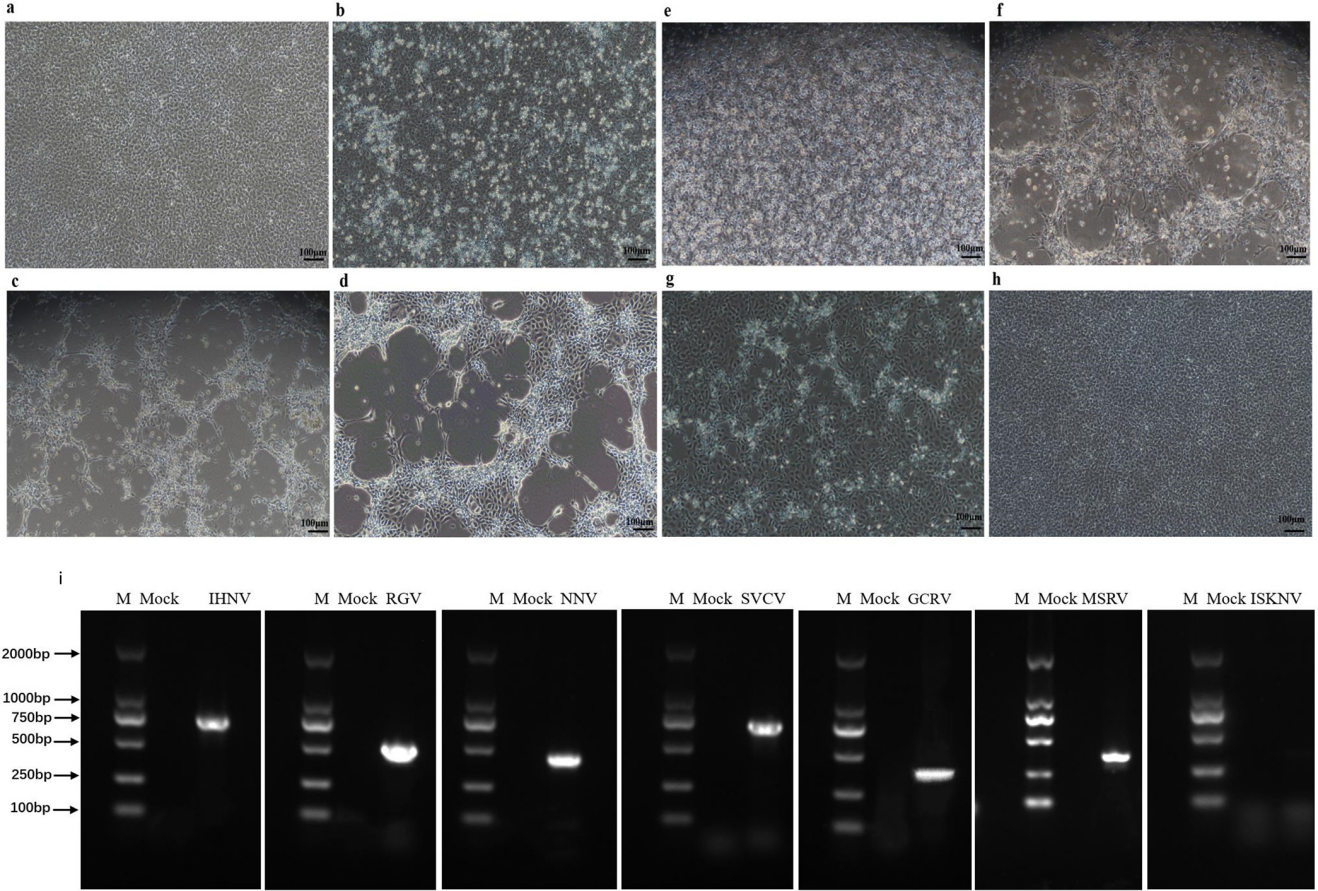
Multiple fish viruses replicated efficiently in *Micropterus salmoides* brain cells. (a) Mock-infected *M. salmoides* brain cells (bar, 100 μm). (b) *M. salmoides* brain cells infected with *M. salmoides* rhabdovirus (MSRV) at 72 h postinfection. (c) *M. salmoides* brain cells infected with carp sprivivirus (SVCV) at 24 h postinfection. (d) *M. salmoides* brain cells infected with infectious hematopoietic necrosis virus (IHNV) at 24 h postinfection. (e) *M. salmoides* brain cells infected with Rana grylio virus (RGV) at 72 h postinfection. (f) *M. salmoides* brain cells infected with Grass carp reovirus (GCRV) at 72 h postinfection. (g) *M. salmoides* brain cells infected with red grouper nervous necrosis virus (RGNNV) at 72 h postinfection. (h) *M. salmoides* brain cells infected with infection spleen and kidney necrosis virus (ISKNV) at 72 h postinfection (bar, 100 μm). (i) Detection of viral genome in infected *Micropterus salmoides* brain cells. Line M, DL2000 bp DNA ladder; Mock, uninfected by the virus; MSBr cells infected by infectious hematopoietic necrosis virus, Rana grylio virus, Red grouper nervous necrosis virus, carp sprivivirus (SVCV), grass carp reovirus, *M. salmoides* rhabdovirus (MSRV), and infection spleen and kidney necrosis virus were shown from left to right.

The susceptibility of MSBr cells to various fish viruses was evaluated. Cytopathic effect (CPE) was observed at 24 to 72 h postinfection when MSBr cells were infected with MSRV, carp sprivivirus (SVCV), grass carp reovirus (GCRV), Rana grylio virus (RGV), red grouper nervous necrosis virus (RGNNV), infectious hematopoietic necrosis virus (IHNV), and infectious spleen and kidney necrosis virus (ISKNV) (Fig. 3). CPE caused by SVCV, GCRV, and IHNV initially appeared as localized areas of rounded and refractile cells that later spread over the monolayer in 24 to 72 h to form a network of degenerating cells. The CPE elicited by RGNNV, MSRV, and RGV firstly showed rounded, granular, and refractive cells in localized areas that unfolded throughout the cell layer over another 3 days until all cells degenerated and detached (Fig. 3b, e, and g). No apparent change was observed in ISKNV-infected or mock-infected MSBr cells (Fig. 3a and h). PCR or reverse transcriptase PCR (RT-PCR) was also conducted to determine the viral susceptibility of MSBr cells with virus-specific primer pairs (Fig. 3i). Titration assay indicated the highest titer was for GCRV and SVCV with 10^7.78^ and 10^6.85^ 50% tissue culture infective dose (TCID_50_) mL^−1^, respectively. The virus titers of MSRV, RGV, and RGNNV were 10^6.04^ TCID_50_ mL^−1^, 10^6.70^ TCID_50_ mL^−1^, and 10^7.39^ TCID_50_ mL^−1^, respectively. While the lowest titer of IHNV with 10^5.83^ TCID_50_ mL^−1^ in MSBr cells was observed (see Table S2 in the supplemental material).

### Pathophysiology of the MSBr cells response to MSRV infection.

**FIG 4:**
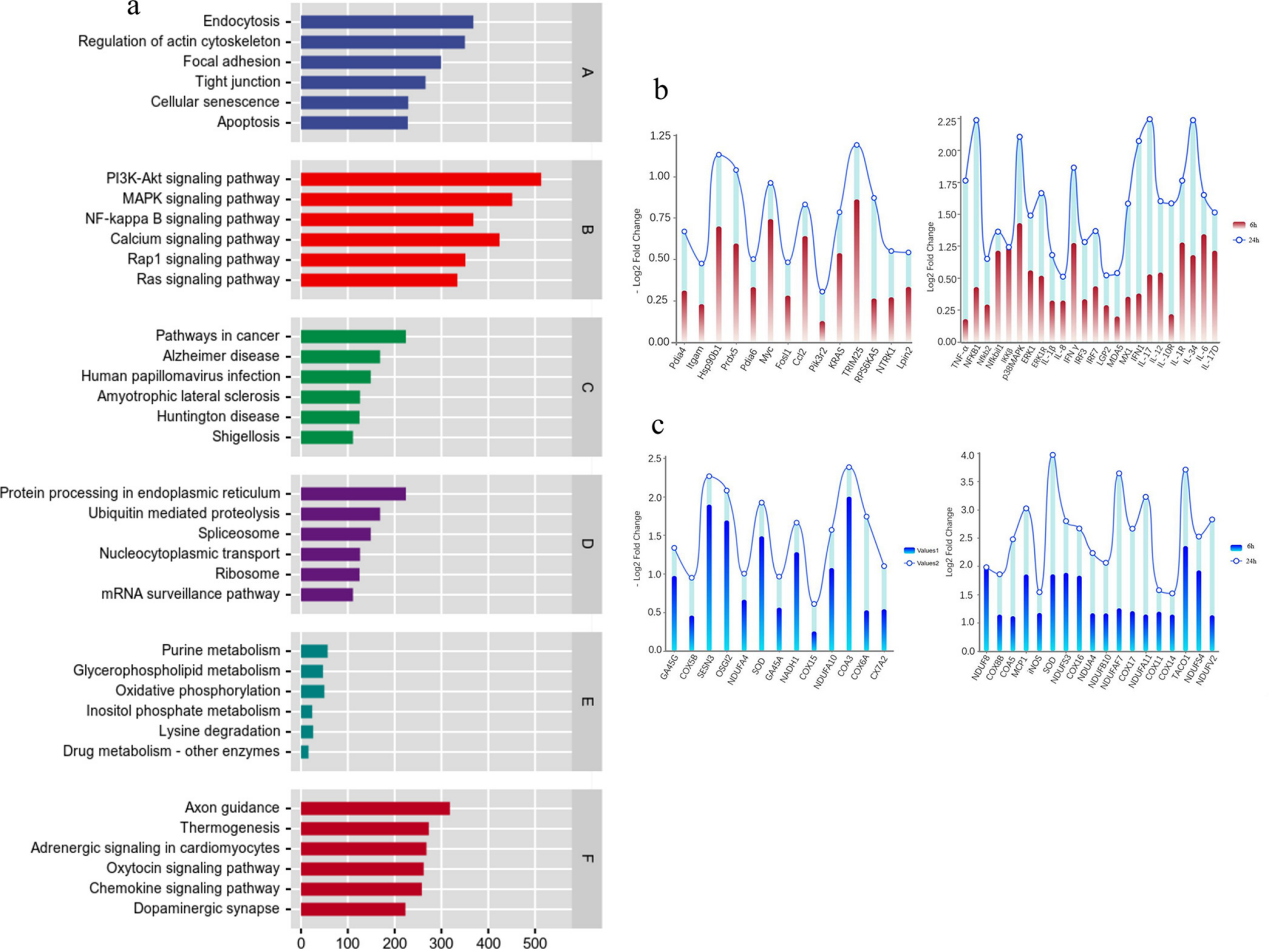
Transcriptome analysis of *Micropterus salmoides* brain cells (MSBr) after MSRV infection. (a) KEGG classification of the MSBr after MSRV infection transcriptome. The *x* axis indicates the number of differentially expressed genes (DGEs). DGEs between different samples were defined if |log2FoldChange| > 1 and *P* < 0.05. The *y* axis indicates the KEGG pathways, and letters A to F represent networks of cellular processes (A), environmental information processing (B), human diseases (C), genetic information processing (D), metabolism (E), and organismal systems (F) signaling pathways. (b) DEGs concerned with inflammation downregulated genes (left) and upregulated genes (right) upon MSRV infection after 6 h or 24 h were shown. (c) DEGs involved in oxidative stress downregulated genes (left) and upregulated genes (right) upon MSRV infection after 6 h or 24 h were shown. Value 1 indicates the sample collected at 6 h, and value 2 indicates the sample collected at 24 h.

To explore the pathophysiology of the MSBr cells response to MSRV infection, MSBr cells harvested from mock- or MSRV-infected groups (for 6 h and 24 h) were subjected to transcriptome sequencing. On average, 360,649,666 clean reads were obtained in the mock-infected and MSRV-infected groups. After MSRV infection, a total of 7,123 genes were differentially expressed between the two groups, including 3,744 upregulated genes and 3,779 downregulated genes (see Fig. S3 in the supplemental material). Among these differentially expressed genes (DEGs), the expression levels of 4,366 genes were increased more than 4 times and 1,711 genes were increased more than 16 times. The most regulated genes including tumor necrosis factor alpha (TNF-α), IkappaB kinase beta (IKKβ), cytochrome *c* oxidase assembly factor 3 (COA3), and sestrin 3 (SESN3), which are associated with inflammatory response and oxidative stress, were changed more than 4 times. GO annotation for all DEGs indicated that 753 GO terms were obtained, which were predominantly comprised of catalytic activity, cellular process, molecular function, and cellular metabolic process. The top 20 levels of GO terms are shown in Fig. S4 in the supplemental material. KEGG analysis on the DEGs indicated that the significantly enriched pathways included regulation of actin cytoskeleton, PI3K-Akt signaling pathway, NF-κB signaling pathway, protein processing in endoplasmic reticulum, chemokine signaling pathway, and others (*P* < 0.05) (Fig. 4a). In detail, inflammation-related genes, such as TNF-α, interleukin 1β, NF-κB1, NF-κB2, IKKβ, and gamma interferon (IFN-γ) were upregulated remarkably after MSRV infection. Notably, 31 DEGs involved in oxidative stress were observed upon MSRV infection, including 13 downregulated genes and 18 upregulated genes. The downregulated genes included several subunits of the NADH dehydrogenase 1 (NADH1), ubiquinone oxidoreductase complex assembly factor 10 (NDVFA10), and ubiquinone oxidoreductase complex assembly factor 4 (NDVFA4), which functions as the first enzyme complex in the electron transport chain. The upregulated genes included superoxide dismutase (SOD), nitric oxide synthase (iNOS), and translational activator of cytochrome *c* oxidase 1 (TACO1), which are known as the stress-induced proteins or damage related molecules (Fig. 4b). Taken together, inflammatory response and oxidative stress may be involved in MSRV-induced pathogenesis.

### Mangiferin and taurine ameliorate MSRV infection.

**FIG 5:**
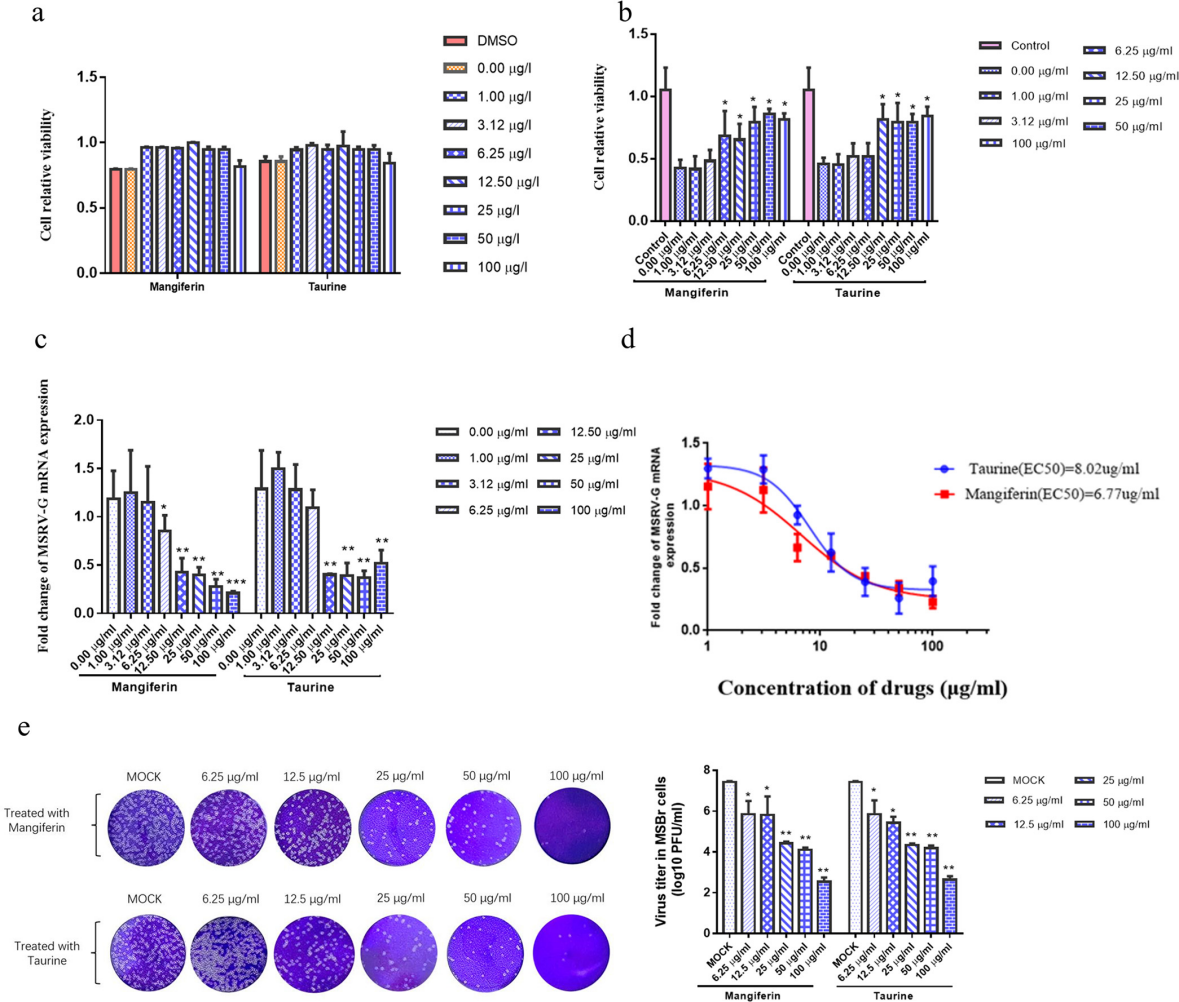
Mangiferin and taurine ameliorate MSRV infection. (a) Cell viability of cells treated with 0- to 100-μg/mL concentrations of mangiferin or taurine. (b) The cell relative viability study of control group (without MSRV infection), 0.00-μg/mL group (with MSRV infection but no mangiferin or taurine treatment), and cells treated with varying concentrations of mangiferin or taurine (1.00, 3.12, 6.25, 12.5, 25, 50, 100 μg/mL) via MTT assay. (c) RT-qPCR analysis of the mRNA expression of MSRV-G in the *Micropterus salmoides* brain cells after mangiferin and taurine treatment. (d) Red and blue represent the viral inhibition curves by linear regression in *M. salmoides* brain cells after mangiferin and taurine treatment, respectively. The EC_50_ values of mangiferin and taurine were 6.77 μg/mL and 8.02 μg/mL, respectively. Data were presented as the mean of triplicates ± SD of duplicate independent experiments, and two-tailed Student's *t* test was performed for statistical analysis. *, *P < *0.05 and **, *P < *0.01 indicate significant difference. (e) Virus titers in the culture supernatants were detected using the viral plaque assay. The culture supernatants were harvested after MSBr cells treated with mangiferin or taurine 8 h and infected with 0.1 MOI MSRV for 24 h. All data are representative of three independent experiments.

Mangiferin and taurine, known as antioxidants, were incubated with the MSBr cells to explore their antiviral capability against MSRV due to the pathophysiology of the MSBr cells response to MSRV. As shown in Fig. 5a, mangiferin and taurine concentrations of 0 to 100 μg/mL had no effect on the activity of MSBr cells. It was observed that treatment of 6.25 μg/mL mangiferin and 12.5 μg/mL taurine exhibit 26.00% and 35.7% cell death, respectively (Fig. 5b). As shown in Fig. 5c, mangiferin inhibited the transcription of MSRV-G by 93.91%, 89.42%, 78.72%, and 61.0% at concentrations of 100, 50, 25, and 12.5 μg/mL, with a slightly dose-dependent manner. Taurine inhibited the replication of MSRV-G by 34.46%, 59.16%, 72.88%, and 58.58% at concentrations of 12.25, 25, 50, and 100 μg/mL, respectively. The 50% effective concentration (EC_50_) values of mangiferin and taurine based on the replication of MSRV were calculated to be 6.77 μg/mL and 8.02 μg/mL, respectively (Fig. 5d). Plaque assay indicated that mangiferin and taurine also inhibited the production of viral particles in the MSBr cells at a low concentration of 6.25 μg/mL. Virus titers in the 6.25 μg/mL, 12.5 μg/mL, 25 μg/mL, and 50 μg/mL treated cells decreased to 5.86, 5.81, 4.43, 4.10, and 2.53 log_10_ PFU/mL, respectively. Notably, for the 100-μg/mL taurine and mangiferin treatment group, the titers of MSRV were 2.53 log_10_ PFU/mL and 2.65 log_10_ PFU/mL, respectively, while the mock group was 7.42 log_10_ PFU/mL (Fig. 5e). In total, we found that mangiferin and taurine cloud ameliorate MSRV infection.

### Mangiferin and taurine attenuate MSRV-induced inflammation.

**FIG 6:**
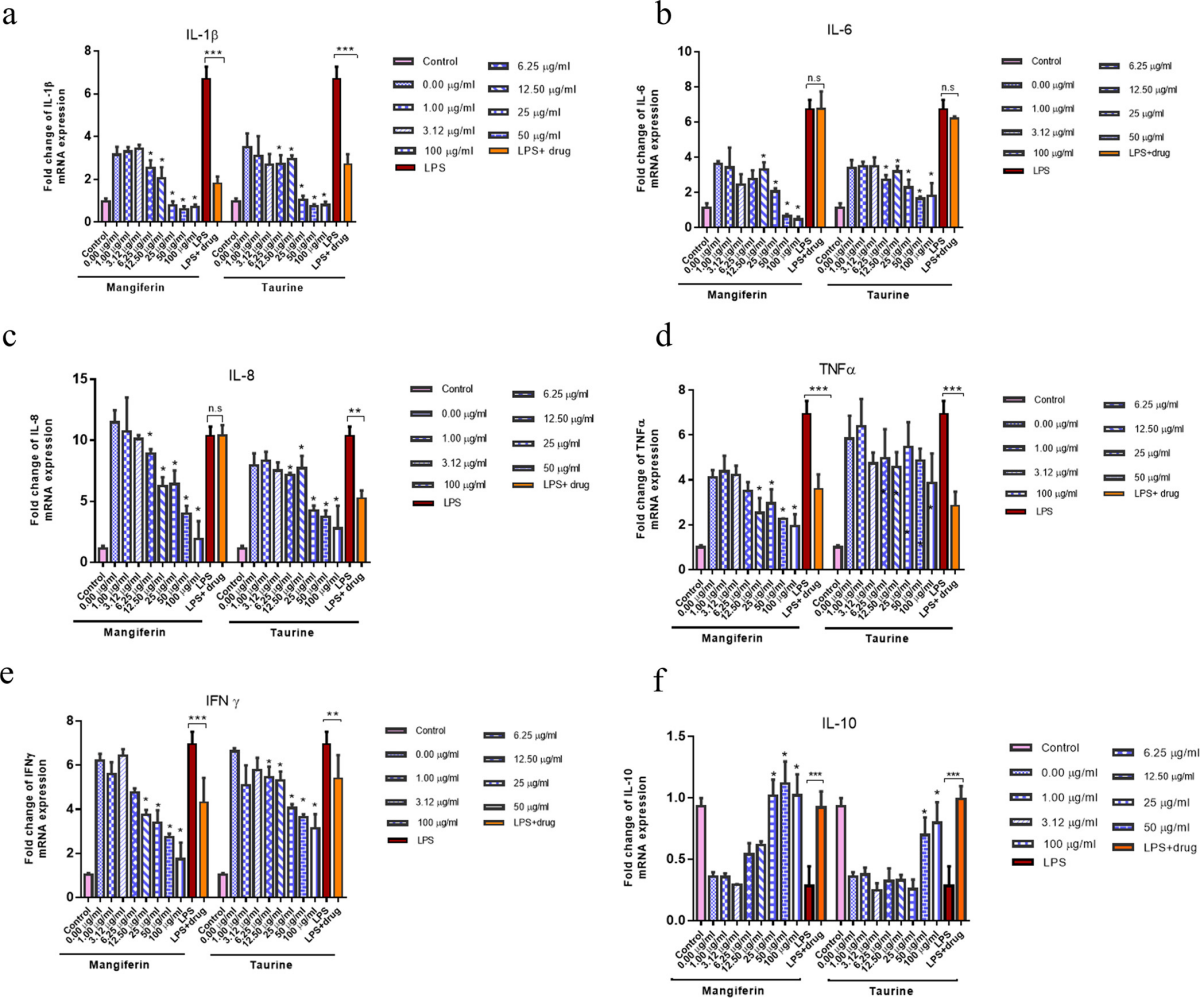
The mRNA expression profile of inflammation-related genes in *Micropterus salmoides* brain cells (MSBr) after MSRV infection. The cells were pretreated with 0- to 100-μg/mL concentrations of mangiferin or taurine and then infected with 0.1 MOI MSRV or LPS (5 μM) for 24 h. (a to f) RT-qPCR analysis of the mRNA expression of interleukin-1β (IL-1β), interleukin-6 (IL-6), interleukin-8 (IL-8), tumor necrosis factor α (TNF-α), interferon γ (IFN-γ), and interleukin-10 (IL-10) in the *M. salmoides* brain cells. LPS^+^ drug indicates the sample collected from the group, in which cells were pretreated with 100-μg/mL concentrations of mangiferin or taurine and then infected with LPS (5 μM) for 24 h. All data are representative of three independent experiments. Data are expressed as the mean ± SD, and two-tailed Student's *t* test was performed for statistical analysis. *, *P < *0.05; **, *P < *0.01; ***, *P < *0.001 indicate significant difference; ns, no difference.

Inflammatory cytokines were detected using reverse transcriptase quantitative PCR (RT-qPCR) assay to explore the antiviral mechanism of mangiferin and taurine. The mRNA level of interleukin-1β (IL-1β) was significantly increased by more than 3.29-fold in MSRV-infected cells compared to that in mock-infected cells (3.14 ± 0.38 versus 0.96 ± 0.15; *P *< 0.05). However, it was blunted to the baseline level when MSBr cells were treated with mangiferin or taurine prior to infection with MSRV (0.89 ± 0.03 in mangiferin group versus 0.97 ± 0.03 in taurine group) (Fig. 6a). Likewise, the IL-6, IL-8, TNF-α, and IFN-γ level was increased approximately 3.29- to 10.68-, 4.15-, and 6.049-fold, respectively, after MSRV infection and returned to a level comparable to the control after mangiferin or taurine treatment (Fig. 6b to e). Furthermore, MSRV infection significantly decreased the level of IL-10 by approximate 2-fold, but mangiferin or taurine significantly rescued the MSRV-induced reduction in IL-10 in MSBr cells (Fig. 6f). Consistently, mangiferin and taurine showed the similar anti-inflammation properties in the presence of lipopolysaccharide (LPS). These observations demonstrated that MSRV infection caused inflammation while mangiferin and taurine attenuated MSRV-induced inflammation.

### Mangiferin and taurine treatment rescued the oxidative stress caused by MSRV infection.

**FIG 7:**
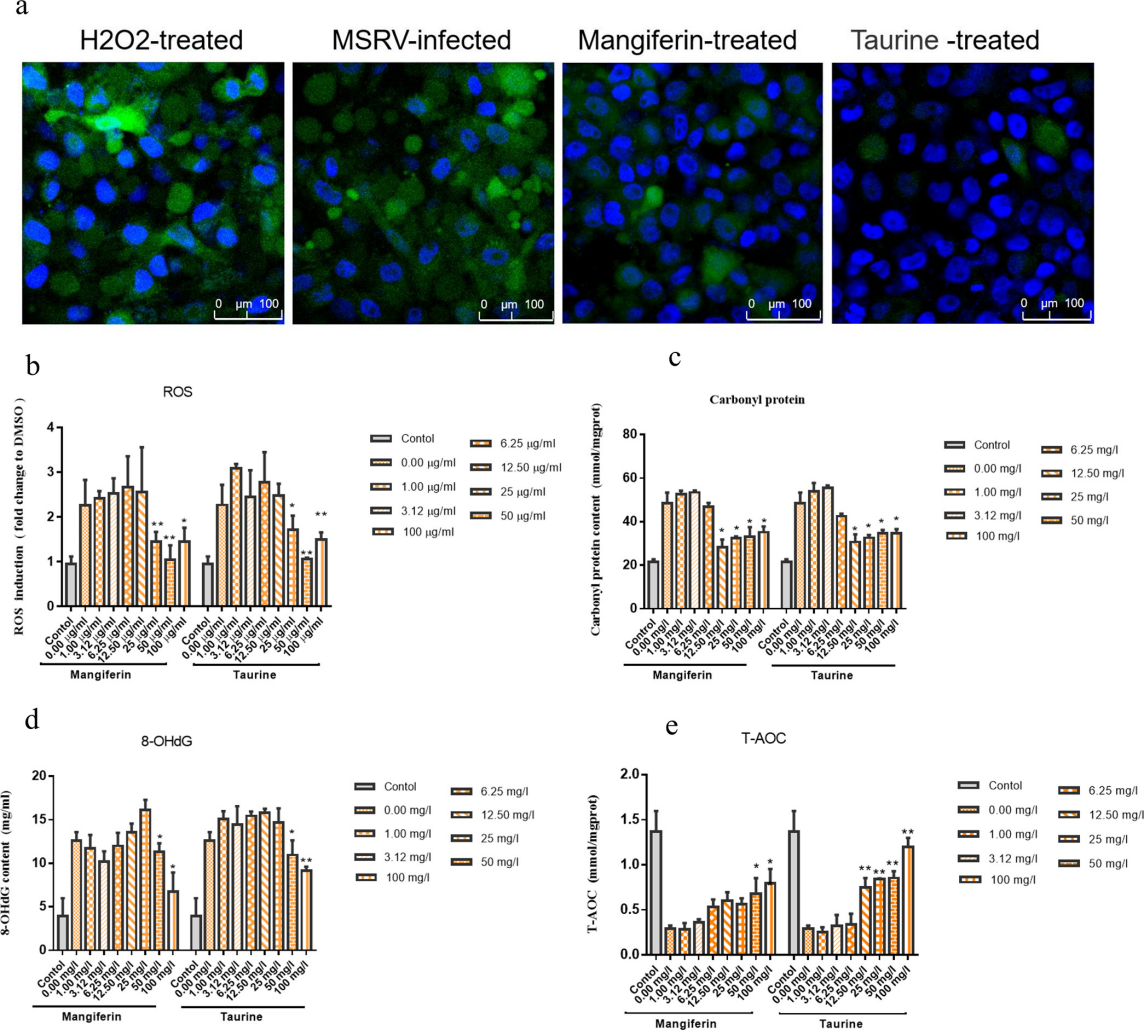
The antioxidative activity of mangiferin and taurine during MSRV infection. (a) ROS levels in H_2_O_2_-treated cells, MSRV-infected cells, and mangiferin- or taurine-treated cells were measured via FV3000 fluorescence microscope. Scale bar of images, 100 μm. (b) The ROS levels in control and mangiferin- or taurine- treated during MSRV infection measured by microplate system. (c to e) *Micropterus salmoides* brain cells were harvested with MSRV infection (MOI of 0.1) or mangiferin- or taurine-treated for 8h before MSRV infection to measure oxidative damage stress biomarker carbonyl protein (c), 8-hydroxy-2′-deoxyguanosine (8-OHdG) (d), and total antioxidant capacity (T-AOC) (e). All data are representative of three independent experiments. Data are expressed as the mean ± SD, and two-tailed Student's *t* test was performed for statistical analysis. *, *P* < 0.05 and **, *P* < 0.011 indicate significant difference.

To examine the antioxidative activity of mangiferin and taurine during MSRV infection, the levels of reactive oxygen species (ROS) in MSRV-infected and mangiferin- or taurine-treated cells were measured. The intracellular ROS levels increased significantly in the MSRV-infected and H_2_O_2_-treated cells and decreased significantly on treatment with mangiferin or taurine as shown in Fig. 7a. Further, the quantification of fluorescence intensity showed that the level of ROS was significantly increased by more than 2.41-fold in the MSRV-infected group compared to that in the control group (2.27 ± 0.05 versus 0.94 ± 0.25; *P* < 0.05) but was restored to normal levels after mangiferin or taurine treatment (1.45 ± 0.22 in the mangiferin group versus 1.71 ± 0.09 in the taurine group) in 25 μg/mL (Fig. 7b). The levels of oxidative damage biomarkers, including protein carbonyls and 8-OHdG, were significantly higher in the MSRV-infected cells than in the control cells, while total antioxidant capacity (T-AOC) levels decreased. These indicators were rescued by the treatment of mangiferin or taurine. The contents of ROS, protein carbonyls, and 8-OHdG in the mangiferin or taurine treatment group were decreased significantly compared to those of the MSRV-infected group (*P* < 0.01) (Fig. 7c to e). Compared with the MSRV-infected group, the T-AOC levels were increased by approximately 2.72-fold and 4.13-fold after mangiferin or taurine treatment in 100 μg/mL, respectively (Fig. 7e). Accordingly, these findings demonstrated that MSRV infection caused oxidative stress, and mangiferin or taurine treatment ameliorated oxidative stress in the MSBr cells.

### Mangiferin and taurine suppressed NF-κB activities during MSRV infection.

**FIG 8:**
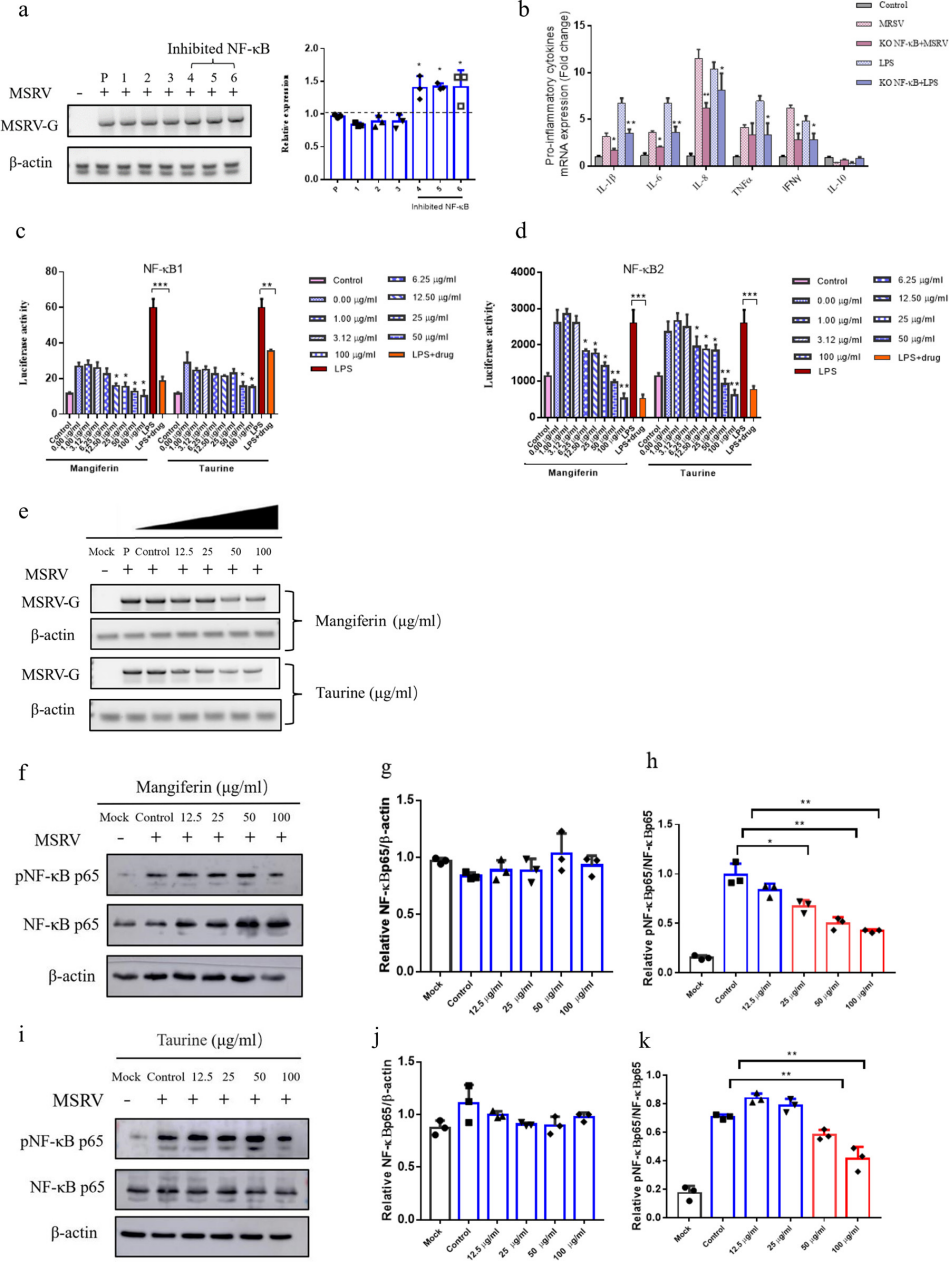
Mangiferin and taurine suppresses NF-κB-1 and NF-κB-2 promoter activities and NF-κB phosphorylation in *Micropterus salmoides* brain cells (MSBr). (a) The mRNA expression of MSRV-G in the infected *Micropterus salmoides* brain cells at 24 h postinfection via semiquantitative RT-PCR (left) and the gray analysis of each group (right). P, positive control. (1 to 3) The mRNA level of MSRV-G in the *Micropterus salmoides* brain cells infected with MSRV at 0.1 MOI. (4 to 6) The mRNA level of MSRV-G in the *Micropterus salmoides* brain cells with inhibition of NF-κB. (b) MSBr cells were pretreated with PDTC (5 μM) for 1 h and then infected with 0.1 MOI MSRV for 24 h. The mRNA levels of IL-1β, IL-6, IL-8, TNF-α, IFN-γ, and IL-10 were determined by RT-qPCR. (c and d) The NF-κB-1 (c) and NF-κB-2 (d) promoter activities. The MSBr cells were transfected with an expression vector for NF-κB-1-Luc or NF-κB2-Luc plus the internal control vector pRL-TK. Then, the cells were infected with 0.1 MOI MSRV for 12 h. MSBr cells in the experimental group were treated with various concentrations mangiferin or taurine for 8 h prior to performing operations of the above. (e) The mRNA level of MSRV-G in the mock (without MSRV infection), control cells (with MSRV-infection but no mangiferin or taurine treatment), and cells treated with various concentrations mangiferin. (f to h) The expression of phosphorylation NF-κBp65 (p NF-κBp65). NF-κBp65 was determined by Western blotting analysis in control cells (without mangiferin treatment) and cells treated with various concentrations mangiferin. (i to k) The expression of the phosphorylated NF-κBp65 (pNF-κBp65); NF-κBp65 was determined by Western blotting analysis in control cells (without taurine treatment) and cells treated with various concentrations taurine. Data are expressed as the mean ± SD, and two-tailed Student's *t* test was performed for statistical analysis. *, *P < *0.05; **, *P < *0.01; and ***, *P < *0.001 indicate significant difference. The red bars represent the significant difference between the experimental group and the control group.

**FIG 9:**
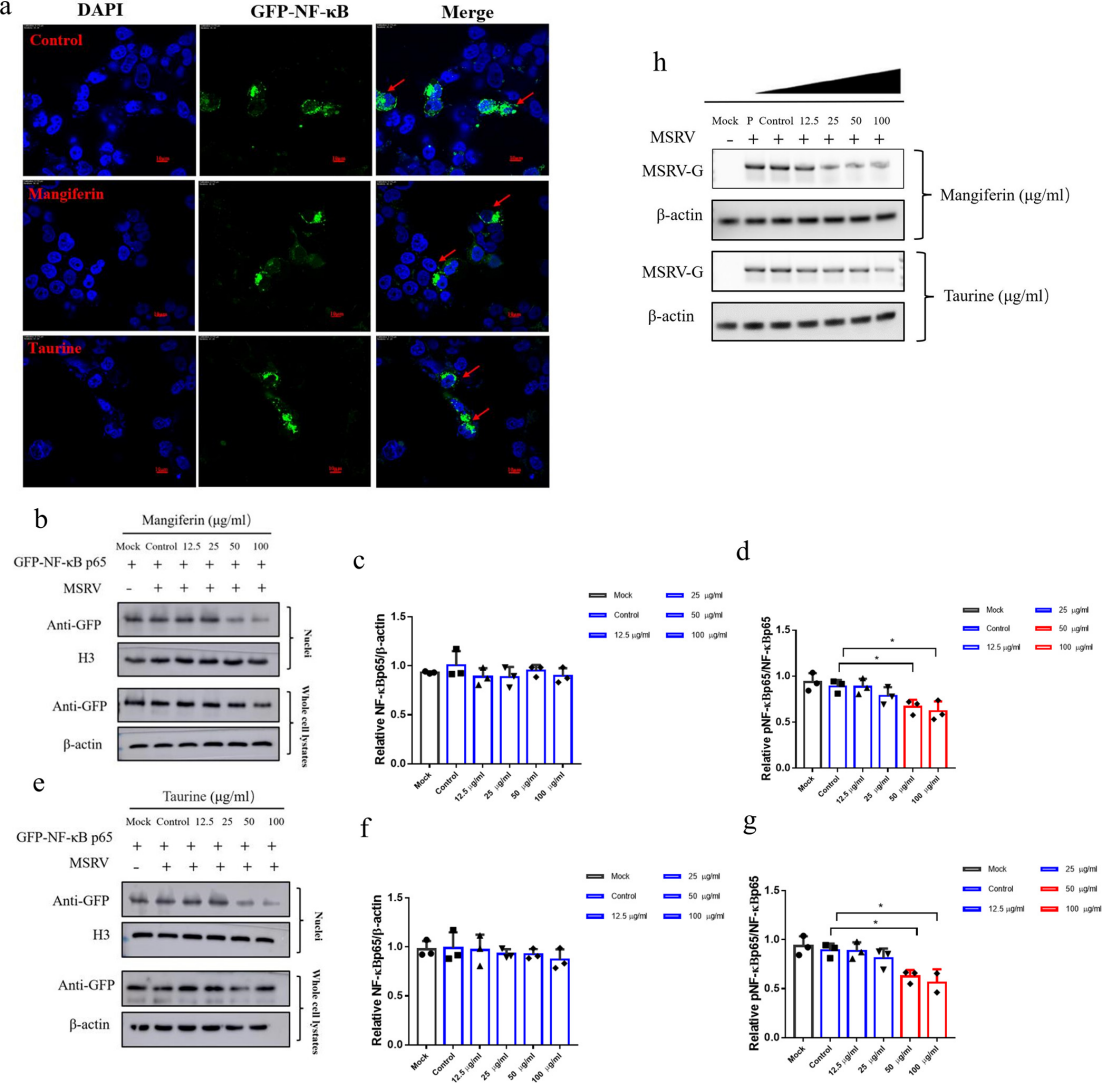
Mangiferin and taurine inhibited NF-κBp65 translocation in *Micropterus salmoides* brain cells (MSBr). (a) MSBr cells were transfected with pEGFP-N1- NF-κBp65 12 h prior to treating with 50 μg/mL mangiferin or taurine for 8 h. Then, the cells were infected with 0.1 MOI MSRV for 12 h. NF-κB p65 translocation was determined by immunofluorescence assay. Scale bar, 100 μm. (b to d) The translocation of NF-κB (p65) from cytoplasm into nucleus in mangiferin-treated groups were determined by Western blotting analysis and quantitative analysis. (e to g) The translocation of NF-κB (p65) from cytoplasm into nucleus in taurine-treated groups was determined by Western blotting analysis and quantitative analysis. (h) The mRNA level of MSRV-G in the mock (without MSRV infection), control cells (with MSRV infection but no mangiferin or taurine treatment), and cells treated with various concentrations of mangiferin. The red bars represent the significant difference between the experimental group and the control group.

NF-κB inhibitors (PDTC) were used to clarify the exact pathway of the MSRV-induced inflammatory response. As expected, inhibition of the NF-κB pathway significantly decreased the expression of IL-1β, IL-6, IL-8, TNF-α, and IFN-γ (47.50%, 44.70%, 46.16%, 19.04%, and 54.08%, respectively) and increased the expression of IL-10 by 1.82-fold in MSRV-infected cells compared with that in the control MSRV-infected group (Fig. 8a and b). To investigate whether the NF-κB pathway is implicated in the antiviral activity of mangiferin and taurine, the promoter activity of NF-κB1 (p50) and NF-κB2 (p52) after MSRV infection and mangiferin or taurine treatment were measured through luciferase activity assays. Compared with cells without infection, MSRV infection caused significant increases of NF-κB1 or NF-κB2 promoter activities by approximately up to 2.29-fold and 2.36-fold, respectively. Moreover, the promoter activities of NF-κB1 and NF-κB2 restored the cytoplasm following mangiferin or taurine treatment (Fig. 8c and d). As shown in Fig. 8f to h, mangiferin or taurine treatment ameliorated the phosphorylated NF-κB (p65) protein level. Interestingly, the level of total NF-κB (p65) protein did not change significantly, which was not consistent with the observations of NF-κB1 or NF-κB2 promoter activities. Furthermore, the translocation of NF-κB (p65) from cytoplasm into nucleus were detected via immunofluorescence assay. The results indicated that mangiferin or taurine treatment resulted in suppressed NF-κB (p65) fluorescence staining in the nucleus, indicating the decreased nuclear translocation of NF-κB (p65) (Fig. 9a). Western blot analysis and quantitative analysis of NF-κB (p65) in the nuclei from each group further validated that mangiferin or taurine treatment consistently suppressed the translocation of NF-κB (p65) from cytoplasm into nucleus (Fig. 9b to g), while mangiferin or taurine treatment caused significant decreases of the mRNA level of MSRV-G (Fig. 8e and Fig. 9h), which was consistent with previous observations (Fig. 5c). These data were conclusive that mangiferin or taurine protected against MSRV-induced inflammation and oxidative stress via suppressing NF-κB signaling.

## DISCUSSION

Understanding the pathogenic mechanism of viral agents and developing effective antivirals could contribute to fish and shellfish production. In this study, based on a novel cell line derived from the brain of *M. salmoides* (MSBr), MSRV-induced inflammatory and oxidative stress responses were confirmed. Consequently, mangiferin and taurine were found to inhibit MSRV infection by blocking NF-κB signaling, representing a kind of BSA with the aim to dampen the inflammatory and oxidative stress responses.

Cell lines are essential for the isolation, proliferation, and characterization of viruses as well as pathogenesis research and drug screening. Currently, more than 600 fish cell lines were recorded in the Cellosaurus cell line database (https://web.expasy.org/cellosaurus/) (21). The MSBr cell line, identified as epithelial cells, was confirmed to be susceptible to MSRV, SVCV, GCRV, RGV, RGNNV, and IHNV, suggesting the potentiality for isolating and identifying infectious agents from *M. salmoides* or others. It is interesting to find that MSBr cells can support the proliferation of GCRV and RGNNV. GCRV mainly infects grass carp and other cyprinid fish and causes severe economic losses in Asia (22–23). RGNNV mainly infects grouper, other marine fishes, and a few freshwater fishes; causes mass economic losses to aquaculture; and serves as one of the most important worldwide pathogens in the larval rearing of several seawater species (24–26). The polyculture of *M. salmoides* and other species or sharing ponds and other aquaculture facilities might cause interspecies transmission of GCRV or RGNNV. Despite no case being reported in the *M. salmoides* culture industry worldwide, the susceptibility and potential threat of GCRV or RGNNV have yet to be assessed.

Elucidating the host-virus interaction provides potential targets for antiviral screening. Transcriptome analysis and biochemical tests indicated that MSRV triggered multiple pathways implicated in inflammation and oxidative stress. As studied in SARS-CoVs, the spike protein binds to TLR2 and TLR1/6 and activates the adaptor protein MyD88, which in turn activates NF-κB to regulate the expression of proinflammatory cytokines (27). Several studies also pinpointed that rabies virus (RABV), a member from the family of *Rhabdoviridae*, induced inflammatory response via NF-κB and mitogen-activated protein kinase (MAPK) (28). Our results showed here that MSRV could activate the promoters of NF-κB1/NF-κB2 and enhance the phosphorylation of p65 and, subsequently, the contents of the nucleus. These findings indicated that the NF-κB pathway involved the inflammatory response induced by MSRV infection in MSBr cells. However, the detail mechanism derived from virus and host for MSRV-mediated inflammatory response needs further investigation. Another unresolved question is the link between inflammatory and oxidative stress during MSRV infection. Accumulating reports demonstrated that hepatitis C virus (HCV), SVCV, and many other viruses caused ROS accumulation and thus triggered an inflammatory response through the NF-κB or MAPK pathway. Taking into consideration the increase of ROS as well as the oxidative damage biomarkers (protein carbonyl and 8-OHdG), we suspected that ROS accumulation during MSRV infection is associated with the inflammatory response and eliminating ROS via antioxidants may relieve the inflammatory response.

Our and other studies showed that mangiferin and taurine exhibit good inhibitory activity against SVCV, poliovirus (PV), herpes simplex virus 1 (HSV-1), and HIV-1 (29–31). Mangiferin and taurine were found to inhibit MSRV infection with the EC_50_ values of 6.77 μg/mL and 8.02 μg/mL, respectively. In addition to their antioxidant activity, a growing body of literature shows the anti-inflammatory properties of mangiferin and taurine (32). Our data demonstrated that mangiferin and taurine significantly decreased the levels of ROS and increased the levels of T-AOC in MSBr cells after MSRV infection (Fig. 7). Mangiferin and taurine also showed their anti-inflammatory properties by dampening the expression of proinflammatory cytokines after MSRV infection (Fig. 6). It is an interesting question whether their antiviral activity of mangiferin and taurine depends on their antioxidant and/or anti-inflammatory capability. Mangiferin and taurine play a certain protective role via the activation of the nuclear factor E2-related factor 2 (Nrf2), a crucial transcription factor that regulates the basal and inducible expression of many antioxidant response element (ARE)-dependent genes (33). Ample evidence also indicated the association between Nrf2 and NF-κB signaling pathways (34). We speculated that mangiferin and taurine could inhibit the NF-κB signaling by upregulating Nrf2, which were utilized by another antioxidant, chlorogenic acid. While mangiferin was reported to regulate NF-κB and prevents inflammatory disorders through the inhibition of IRAK1 phosphorylation (35). The elaborate association between Nrf2 and NF-κB signaling pathways, representing the key cellular defense response of antioxidant and anti-inflammatory response, need further investigation. The answer will contribute to the development of BSAs.

In conclusion, inflammatory response and oxidative stress involved the MSRV-induced pathogenesis. Mangiferin and taurine inhibited MSRV infection by dampening the expression of proinflammatory cytokines and ameliorating oxidative stress, representing a promising BSA.

## MATERIALS AND METHODS

### Fish, infectious agents, and drugs.

*M. salmoides*, approximately 10 cm long, was obtained from a commercial fish farm (Yi-Chen, Hubei, China). These fish were temporarily housed in quarantine tanks at 23 to 25°C with a natural photoperiod for 14 days.

*Micropterus salmoides* rhabdovirus (MSRV), carp sprivivirus (SVCV), grass carp reovirus (GCRV), Rana grylio virus (RGV), red grouper nervous necrosis virus (RGNNV), infectious hematopoietic necrosis virus (IHNV), and infectious spleen and kidney necrosis virus (ISKNV) were used for virus susceptibility investigation. All of them were stored in small aliquots at −80°C. Mangiferin and taurine were purchased from Sigma-Aldrich.

### Primary culture preparation.

The fish were sacrificed with 3-aminobenzoic acid ethyl ester methanesulfonate (MS-222; Sigma-Aldrich) at 4.5 g/L and then swabbed with 75% alcohol. The brain was removed and washed 3 times with phosphate-buffered saline (PBS) (pH = 7.4), and then the brain tissue was soaked in Hanks’ balanced salt solutions (HBSS) containing 200 IU/mL penicillin and 200 IU/mL streptomycin for 30 min. The brain was removed, finely cut with scissors, and transferred to a trypsinization flask with 3 mL of 0.25% trypsin-EDTA resolution (Gibco, Carlsbad, CA). The process was performed at 28°C for 20 min, and then, the trypsinized cells were filtered through 70-μm cell strainers. The resultant cells were centrifuged at 1,000 × *g* for 7 min. The cell pellet was suspended in Medium 199 with 20% fetal bovine serum (FBS) (Gibco, Carlsbad, CA) seeded into 25-cm^2^ tissue culture flasks and incubated at 28°C.

### Subculture, maintenance, storage, and revival.

When a fused monolayer was formed in the primary culture, it was trypsinized with 0.25% trypsin-EDTA solution. The cells were subcultured at a 1:2 to 1:3 quantitative ratio and maintained within Medium 199 with 20% FBS, 200 IU/mL penicillin, and 200 μg/mL streptomycin for the initial five passages. After five passages, the concentration of FBS in the medium was reduced to 15%. Ten passages later, the concentration of FBS in the medium was reduced to 10%. The subcultures were stored in liquid nitrogen every 5 to 10 passages in freezing medium, which consisted of 90% FBS and 10% dimethyl sulfoxide (DMSO). For revival, a cryovial was thawed quickly in a water bath at 37°C and centrifuged at 1,000 × *g* for 7 min at 4°C. The cells were resuspended in Medium 199 with 20% FBS, 200 IU/mL penicillin, and 200 μg/mL streptomycin and seeded in a 25-cm^2^ tissue culture flask. After three passages, the concentration of FBS in the medium was reduced to 10%. The viability of the revived cells was calculated by a Countstar automatic cell counter.

### Growth studies.

The effects of temperature and FBS concentration on the growth of MSBr cells were analyzed at passage 26. Falcon 12-transwell plates were seeded at 1 × 10^5^ cells per well and incubated at 28°C for 2 h to permit cell attachment. Then, batches of flasks were incubated at selected temperatures of 16°C, 22°C, 28°C, and 37°C for 6 days. Each day, 3 wells of cells at each temperature were washed twice with PBS, and the cell density was measured by an automatic cell counter (Countstar; IC1000). The expansion response to different concentrations of FBS (2%, 5%, 10%, and 15%) was evaluated with a similar procedure at 28°C.

### Chromosome number analysis.

MSBr cells at passage 26 were used for chromosome analysis. Briefly, MSBr cells were cultured in a 25-cm^2^ tissue culture flask until reaching 80 to 90% confluence at 28°C and treated with 15 μg/mL Colcemid (Guiecham; ZY64) for 8 h at 28°C. Then, the cells were washed with PBS, trypsinized with 0.25% trypsin-EDTA, and collected by centrifugation at 1,000 × *g* for 7 min. After resuspension in 6 mL of cold water for 30 min, the cells were fixed in 5 mL of acetic acid/methanol (1:3) for 15 min. The cell suspension was dropped onto a clean precooled microslide (Thermo Fisher Scientific). The cells were stained with Giemsa (G8220; Solarbio, Beijing) for 30 min at room temperature, and a total of 80 chromosome spreads were counted under phase-contrast microscopy.

### DNA (RNA) extraction, PCR, and qPCR analysis.

The DNA of each sample was extracted according to the manufacturer's manual (CW2298; CWBio, China). The total RNA was extracted using TRIzol reagent according to the manufacturer's manual (Vazyme Biotech, China). HiScript reverse transcriptase (Vazyme Biotech, China) was used to process total RNA into cDNA, and PCR was performed in 50 μL of PCR buffer containing 3 μL of DNA (cDNA) template with each primer using the following steps: denaturation at 95°C for 5 min followed by 30 cycles of denaturation at 95°C for 40 s, annealing temperature as shown in Table S1 in the supplemental material for 40 s and elongation at 72°C for 90 s, ending with an additional elongation step of 10 min at 72°C. All of the primer pairs were referred to the previous reports (Table S1). PCR products were analyzed by 1% agarose gel electrophoresis, and the expected PCR products were purified and sequenced. qPCR was carried out in 20-μL reaction mixtures, which contained 10 μL of AceQ qPCR SYBR green master mix and 0.5 μL of each primer. The reactions were performed using the following conditions: 96°C for 3 min; 96°C for 30 s, 56°C for 60 s, 72°C for 90 s, 35 cycles; and 72°C for 10 min.

### Viral susceptibility and viral plaque assay.

The susceptibility of MSBr cells to various fish viruses, including MSRV, SVCV, GCRV, RGV, RGNNV, IHNV, and ISKNV, was determined at the 28th passage as previously described (36). Briefly, cells were seeded into a 6-well plate and incubated for 24 h at 28°C. Then, 0.2 mL of virus (multiplicity of infection [MOI] = 0.1) suspension was inoculated into each 6-well plate and allowed to adsorb for 1 h. Unattached viruses were removed, 2 mL of maintaining medium containing 5% FBS was added to each 6-well plate, and the cells were maintained at 28°C and examined daily for the appearance of cytopathic effects.

For viral plaque assay, MSBr cells, treated with mangiferin or taurine, were infected with MSRV at 0.1 MOI. After 24 h postinfection, culture supernatants were harvested. The viral titers were determined by using the plaque assay according to previous studies (37–38). Specifically, MSBr cells were seeded in 12-well plates and grown to1 × 10^5^/cm^2^. Culture supernatants were treated at 10-fold serial dilutions according to a gradient, after which they were inoculated into monolayers of cells at a volume of 0.1 mL/well. After 2 h of virus attachment, the infection medium was removed, and cells were overlaid with the M199 medium containing 3% carboxymethyl cellulose (CMC) (Sigma-Aldrich) and 5% FBS at 28°C for a period of 72 h. Then, the cells were fixed with 10% formaldehyde for 6 h and stained with 0.5% crystal violet for 1 h. Visible plaques were counted after washing with tap water, and viral titers were calculated by using the formula PFU/mL =10a × b × (1,000/c) (a, dilution times of virus-containing supernatant; b, the number of the plaque; c, the volume of virus-containing supernatant).

### Cell transfection.

MSBr cells at the 28th passage were seeded at a density of 1 × 10^5^ cells well^−1^ in a 6-well plate for the transfection assay. When cells reached subconfluent monolayers, cells were transfected with the pEGFP-N1 plasmid using Fish-Trans (Meisente Biological, Wuhan) and PEI reagent (Zhongshi, Shanghai) according to the manufacturer's instructions. After 24 to 48 h, the green fluorescence signals were observed by Zeiss fluorescence microscopy, and the transfection efficiency was calculated accordingly.

### Western blotting and immunofluorescence.

Cells were collected by cold PBS and lysed on ice in radioimmunoprecipitation assay (RIPA) lysis buffer (Beyotime, P0013), followed by centrifugation at 3,500 × *g* at 4°C for 5 min, to remove cell debris. The protein concentration was tested by bicinchoninic acid (BCA) protein assay kit (Beyotime, P0012) according to the manufacturer’s instructions. Twenty-microgram protein samples were separated by 12% (vol/vol) SDS-polyacrylamide gel and transferred to polyvinylidene difluoride membranes (Bio-Rad; no. 1620177). The membranes were blocked with 5% skimmed milk in Tris-buffered saline supplemented with 0.1% Tween 20 (TBST) (Cell Signaling Technology; no. 9997) at 4°C overnight, and then washed with TBST (TBS containing 0.1% Tween 20) buffer for 3 × 10 min. The membranes were then incubated with primary antibodies against the KRT18 antibody (EnoGene; E1A0191) and the VIM antibody (EnoGene; E1A7013), respectively, for 2 h. After three washes with TBST, the membranes were incubated with peroxidase-conjugated anti-rabbit (KPL; 074–1506) at room temperature for 1 h and then washed with TBST buffer for 3 × 5 min, The epitope was visualized by using an ECL Western blot detection kit (Millipore; WBKLS0500).

For indirect immunofluorescence assay, cells were seeded in chamber slide (Thermo, USA) for 24 h and fixed by 4% paraformaldehyde for 10 min. Then, the cells were permeated with 0.2% Triton X-100 for 20 min. After incubation in TBST containing 2% skim milk for 2 h and three washes with TBST (each 5 min), cells were incubated with the primary antibodies against KRT18 and VIM, respectively, at 4°C, overnight. After washing with TBST three times, the cells were incubated with either Alexa Fluor 568- or Alexa Fluor 488-conjugated secondary antibodies, respectively, for 45 min. The nuclei were stained by 4′,6-diamidino-2-phenylindole (DAPI) (Beyotime; C1006). Original immunofluorescence images were acquired by using an Olympus FluoView 1000 confocal microscope (Olympus) and saved as LIM images (.nd2). Sharpened and merged images were exported and saved as TIF format by using NIS-Elements viewer 5.21 (.tif).

### Transcriptome sequencing and data analysis.

Mock or MSRV-infected (MOI = 0.1) MSBr cells were collected at 6 h and 24 h postinfection, separately, to analyze the expression profiles of host genes involved in MSRV infection. Sequencing libraries were generated using the NEB Next Ultra RNA library prep kit for Illumina (NEB, USA) following the manufacturer’s protocol, and library quality was assessed on the Illumina sequencing platform (HiSeq 2500). Low-quality sequences (Q > 20) were first cleaned by removing adaptor sequences and then mapped to the reference genome (ASM1485139v1). Differential statistical analyses of gene expression were performed using the DESeq2 R package (1.20.0). Benjamini and Hochberg’s approach was used to control *P* values, and genes with an adjusted *P* value if <0.05 found by DESeqs were assigned as differentially expressed genes (DEGs). GO enrichment analyses and KEGG enrichment analyses were performed with the database established by the Gene Ontology Consortium (http://geneontology.org/) and Kyoto Encyclopedia of Genes and Genomes (http://www.kegg.jp/), respectively. The raw data of transcriptomic sequencing have been deposited in the Genome Sequence Archive (accession number PRJNA865434).

### Cell viability assay.

According to a previous description (30), mangiferin and taurine cytotoxicity was determined by a 3-(4,5-di-methylthiazol-2-yl)-2,5-diphenyltetrazolium bromide (MTT) assay kit (Sigma-Aldrich, USA). Specifically, MSBr cells were passaged in 96-well microplates (Corning 3799, USA) and were treated with varying concentrations of mangiferin or taurine (1.00 μg/mL to 100 μg/mL) and incubated at 28°C with 5% CO_2_ for 72 h. The cell viability (%CV) was tested by Varioskan LUX (Thermo Scientific) and calculated with the formula %CV = (*A*t/*A*c) × 100, where *A*t and *A*c refer to the absorbance of mangiferin (or taurine group) and control (untreated cells), respectively.

### The *in vitro* antiviral activity.

MSBr cells at the 35th passage were used to evaluate the antiviral effects of mangiferin and taurine. Briefly, MSBr cells were seeded at a density of 1 × 10^5^ cells well^−1^ in a 6-well plate, grown to confluence and treated with varying concentrations of mangiferin or taurine (1.00, 3.12, 6.25, 12.5, 25, 50, 100 μg/mL). After washing with PBS, 0.2 mL of MSRV suspension at 0.1 MOI was inoculated into the cell culture in each well plate and allowed to adsorb for 1 h at 28°C. After washing away free viruses, complete medium containing the corresponding concentrations of mangiferin and taurine was added to the cells, and the cells were cultured in a 28°C, 5% CO_2_ incubator for 24 h. Three independent repeats were set for each treatment. Twenty-four hours later, the cells were collected to extract total RNA, and the transcribed level of the MSRV-G gene was determined by RT-qPCR with the primer pair (5′-CCGTCCAAACTAGCAACAT-3′, 5′- TGGGTGCCTCACTGGGTAT -3′), and β-actin was used as the reference gene (39). The 50% inhibitory concentration (EC_50_) was determined as the concentration of the compound capable of reducing the virus titer by 50% based on linear regression of the viral inhibition curves.

### Luciferase activity assay.

One hundred nanograms of Renilla luciferase internal control vector (pRL-TK) and 1,000 ng of NF-κB1(p50)-Luc or NF-κB2-(p52)-Luc were transiently transfected in MSBr cells in 12-well plates using fish-trans reagent. After transfection 12 h, the cells were stimulated with mangiferin or taurine for 8 h and were then infected with MSRV at an MOI of 0.1. At 24 h postinfection, cells were washed by PBS. The luciferase activity was measured with the Dual-Luciferase reporter assay system according the manufacturer's instructions (Promega; E1960).

### Measurement of oxidative stress biomarkers.

The determination of intracellular ROS was performed according to previous studies (40). In brief, MSBr cells were seeded at a density of 1 × 10^5^ cells in a confocal dish. The cells were stimulated with mangiferin or taurine for 8 h and were then infected with MSRV at an MOI of 0.1. At 12 h postinfection, cells were washed by PBS, and H2DCFDA (DCFH-DA) was added with the working concentration of 2 mM followed by incubation in the dark at 37°C for 20 min. Cells were washed with PBS three times and stained with DAPI. The fluorescence signal was collected by FV3000 fluorescence microscope. For quantitative assessment, MSBr cells were seeded at a density of 1 × 10^4^ cells in a 96-well plate, and then steps were executed which were roughly as described above, and luminescence signal was measured by microplate reader.

The cells of each group were harvested and lysed by ultrasonication, and after centrifugation at 3,500 × *g* for 10 min, the supernatant was collected for analysis. The level of protein carbonyl, 8-hydroxy-2′-deoxyguanosine (8-OHdG) and total antioxidant capacity (T-AOC) were measured using the 8-OHdG enzyme-linked immunosorbent assay (ELISA) kit (Jiancheng, Nanjing; YB-E4059), Xanthine oxidase activity kit (Solarbio, Qingdao; BC1270), and T-AOC activity kit (Solarbio, Qingdao; BC1315) following the manufacturer’s instructions.

### Statistical analysis.

For all data analyses, GraphPad Prism 7.0 software (GraphPad soft, USA) was used. A *P* value of <0.05 (*) was considered statistically significant, and a *P* value of <0.01 (**) was considered extremely significant. The results were expressed as the mean ± variance (standard deviation [SD]).

### Ethical approval.

All fish used in this study were housed and cared for at HZAU in accordance with Institutional Animal Care and Use Committee guidelines that were approved by the Scientific Ethics Committee of Huazhong Agricultural University. The approval protocol number was HZAUFI-2019-01.

### Data availability.

The data sets generated during and/or analyzed in the current study are available from the corresponding author on reasonable request. The MSBr cell line has been deposited in the China Center for Type Culture Collection (CCTCC no. C202166).

## References

[1] Ampomah PB, Lim LHK. 2020. Influenza A virus-induced apoptosis and virus propagation. Apoptosis 25:1–11. doi:10.1007/s10495-019-01575-3. 31667646

[2] Galindo I, Alonso C. 2017. African swine fever virus: a review. Viruses 9:103. doi:10.3390/v9050103. 28489063 PMC5454416

[3] Khatri R, Parray HA, Agrahari AK, Rizvi ZA, Kaul R, Raj S, Asthana S, Mani S, Samal S, Awasthi A, Ahmed S. 2022. Designing and characterization of a SARS-CoV-2 immunogen with receptor binding motif grafted on a protein scaffold: an epitope-focused vaccine approach. Int J Biol Macromol 209:1359–1367. doi:10.1016/j.ijbiomac.2022.04.148. 35469951 PMC9033297

[4] Lum FM, Torres-Ruesta A, Tay MZ, Lin RTP, Lye DC, Rénia L, Ng LFP. 2022. Monkeypox: disease epidemiology, host immunity and clinical interventions. Nature Rev Immunol 22:597–613. doi:10.1038/s41577-022-00775-4. 36064780 PMC9443635

[5] Kelly RK, Wolf K. 1989. Fish viruses and fish viral diseases. Copeia 1989:821. doi:10.2307/1445539.

[6] Wallace IS, McKay P, Murray AG. 2017. A historical review of the key bacterial and viral pathogens of Scottish wild fish. J Fish Diseases 40:1741–1756. doi:10.1111/jfd.12654. 28718925

[7] El-Atab N, Mishra RB, Hussain MM. 2022. Toward nanotechnology-enabled face masks against SARS-CoV-2 and pandemic respiratory diseases. Nanotechnology 33:062006. doi:10.1088/1361-6528/ac3578. 34727530

[8] Ianevski A, Simonsen RM, Myhre V, Tenson T, Oksenych V, Bjørås M, Kainov DE. 2022. DrugVirus.info 2.0: an integrative data portal for broad-spectrum antivirals (BSA) and BSA-containing drug combinations (BCCs). Nucleic Acids Res 50:W272–W275. doi:10.1093/nar/gkac348. 35610052 PMC9252782

[9] Ianevski A, Andersen PI, Merits A, Bjørås M, Kainov D. 2019. Expanding the activity spectrum of antiviral agents. Drug Discovery Today 24:1224–1228. doi:10.1016/j.drudis.2019.04.006. 30980905

[10] Brai A, Fazi R, Tintori C, Zamperini C, Bugli F, Sanguinetti M, Stigliano E, Esté J, Badia R, Franco S, Martinez MA, Martinez JP, Meyerhans A, Saladini F, Zazzi M, Garbelli A, Maga G, Botta M. 2016. Human DDX3 protein is a valuable target to develop broad spectrum antiviral agents. Proc Natl Acad Sci USA 113:5388–5393. doi:10.1073/pnas.1522987113. 27118832 PMC4868442

[11] Lange CM, Zeuzem S. 2013. Perspectives and challenges of interferon-free therapy for chronic hepatitis C. J Hepatology 58:583–592. doi:10.1016/j.jhep.2012.10.019. 23104162

[12] Lin K, Gallay P. 2013. Curing a viral infection by targeting the host: the example of cyclophilin inhibitors. Antiviral Res 99:68–77. doi:10.1016/j.antiviral.2013.03.020. 23578729 PMC4332838

[13] Ren Q, Ma M, Yang J, Nonaka R, Yamaguchi A, Ishikawa KI, Kobayashi K, Murayama S, Hwang SH, Saiki S, Akamatsu W, Hattori N, Hammock BD, Hashimoto K. 2018. Soluble epoxide hydrolase plays a key role in the pathogenesis of Parkinson's disease. Proc Natl Acad Sci USA 115:E5815–E5823. doi:10.1073/pnas.1802179115. 29735655 PMC6016799

[14] Bikle DD. 2022. Vitamin D regulation of immune function during covid-19. Rev Endocr Metab Disord 23:279–285. doi:10.1007/s11154-021-09707-4. 35091881 PMC8799423

[15] Karin M, Lin A. 2002. NF-kappaB at the crossroads of life and death. Nature Immunology 3:221–227. doi:10.1038/ni0302-221. 11875461

[16] Clapp S. 2009. Aquaculture now accounts for half of all fish consumption. Food Chemical News 28:51.

[17] Crane M, Hyatt A. 2011. Viruses of fish: an overview of significant pathogens. Viruses 3:2025–2046. doi:10.3390/v3112025. 22163333 PMC3230840

[18] Fisheries and Aquaculture Department FAO. 2007. The state of world fisheries and aquaculture. Rome Italy FAO 4:40–41.

[19] Gao EB, Chen G. 2018. Micropterus salmoides rhabdovirus (MSRV) infection induced apoptosis and activated interferon signaling pathway in largemouth bass skin cells. Fish Shellfish Immunol 76:161–166. doi:10.1016/j.fsi.2018.03.008. 29510251

[20] Deng G, Li S, Xie J, Bai J, Chen K, Ma D, Jiang X, Lao H, Yu L. 2011. Characterization of a ranavirus isolated from cultured largemouth bass (Micropterus salmoides) in China. Aquaculture 312:198–204. doi:10.1016/j.aquaculture.2010.12.032.

[21] Ruiz-Palacios M, Almeida M, Martins MA, Oliveira M, Esteban MA, Cuesta A. 2020. Establishment of a brain cell line (fub-1) from mummichog (Fundulus heteroclitus) and its application to fish virology, immunity and nanoplastics toxicology. Sci Total Environ 708:134821. doi:10.1016/j.scitotenv.2019.134821. 31791770

[22] Dong C, Shuang F, Weng S, He J. 2014. Cloning of a new fibroblast cell line from an early primary culture from mandarin fish (Siniperca chuatsi) fry for efficient proliferation of megalocytiviruses. Cytotechnology 66:883–890. doi:10.1007/s10616-013-9642-7. 24101440 PMC4235937

[23] Shen YJ, Xie-Min QI, Liu B, Zhou GH, Tai-Ming LI. 2015. Development and application of a real-time PCR assay for the detection and quantification of Verticillium dahliae. Chinese J Ecol 213:164–173. doi:10.1016/j.jviromet.2014.11.011.

[24] Bandín I, Souto S. 2020. Betanodavirus and VER disease: a 30-year research review. Pathogens 9:106. doi:10.3390/pathogens9020106. 32050492 PMC7168202

[25] Doan QK, Vandeputte M, Chatain B, Morin T, Allal F. 2017. Viral encephalopathy and retinopathy in aquaculture: a review. J Fish Dis 40:717–742. doi:10.1111/jfd.12541. 27633881

[26] Volpe E, Gustinelli A, Caffara M, Errani F, Quaglio F, Fioravanti ML, Ciulli S. 2020. Viral nervous necrosis outbreaks caused by the RGNNV/SJNNV reassortant betanodavirus in gilthead sea bream (Sparus aurata) and European sea bass (Dicentrarchus labrax). Aquaculture 523:735155. doi:10.1016/j.aquaculture.2020.735155.

[27] Khan S, Shafiei MS, Longoria C, Schoggins J, Zaki H. 2020. SARS-CoV-2 spike protein induces inflammation via tlr2-dependent activation of the nf-κb pathway. bioRxiv. doi:10.1101/2021.03.16.435700. PMC8709575 34866574

[28] Liu SQ, Xie Y, Gao X, Wang Q, Zhu WY. 2020. Inflammatory response and MAPK and NF-κB pathway activation induced by natural street rabies virus infection in the brain tissues of dogs and humans. Virology J 17:157. doi:10.1186/s12985-020-01429-4. 33081802 PMC7576862

[29] Rechenchoski DZ, Agostinho KF, Faccin-Galhardi LC, Lonni AASG, da Silva JVH, de Andrade FG, Cunha AP, Ricardo NMPS, Nozawa C, Linhares REC. 2020. Mangiferin: a promising natural xanthone from Mangifera indica for the control of acyclovir-resistant herpes simplex virus 1 infection. Bioorg Med Chem 28:115304. doi:10.1016/j.bmc.2020.115304. 31956052

[30] Rechenchoski DZ, Samensari NL, Faccin-Galhardi LC, de Almeida RR, Cunha AP, Ricardo NMPS, Nozawa C, Linhares REC. 2019. The combination of Dimorphandra gardneriana galactomannan and mangiferin inhibits herpes simplex and poliovirus. Curr Pharm Biotechnol 20:215–221. doi:10.2174/1389201020666190307130431. 30848197

[31] Wang R-R, Gao Y-D, Ma C-H, Zhang X-J, Huang C-G, Huang J-F, Zheng Y-T. 2011. Mangiferin, an anti-HIV-1 agent targeting protease and effective against resistant strains. Molecules 16:4264–4277. doi:10.3390/molecules16054264. 21610656 PMC6263262

[32] Marcinkiewicz J, Kontny E. 2014. Taurine and inflammatory diseases. Amino Acids 46:7–20. doi:10.1007/s00726-012-1361-4. 22810731 PMC3894431

[33] Shao J, Huang J, Guo Y, Li L, Liu X, Chen X, Yuan J. 2016. Up-regulation of nuclear factor e2-related factor 2 (Nrf2) represses the replication of SVCV. Fish Shellfish Immunol 58:474–482. doi:10.1016/j.fsi.2016.09.012. 27693327

[34] Bao L, Li J, Zha D, Zhang L, Gao P, Yao T, Wu X. 2018. Chlorogenic acid prevents diabetic nephropathy by inhibiting oxidative stress and inflammation through modulation of the Nrf2/HO-1 and NF-ĸB pathways. Int Immunopharmacol 54:245–253. doi:10.1016/j.intimp.2017.11.021. 29161661

[35] Jeong JJ, Jang SE, Hyam SR, Han MJ, Kim DH. 2014. Mangiferin ameliorates colitis by inhibiting IRAK1 phosphorylation in NF-κB and MAPK pathways. Eur J Pharmacol 740:652–661. doi:10.1016/j.ejphar.2014.06.013. 24972244

[36] Wen CM, Lee CW, Wang CS, Cheng YH, Huang HY. 2008. Development of two cell lines from Epinephelus coioides brain tissue for characterization of betanodavirus and megalocytivirus infectivity and propagation. Aquaculture 278:14–21. doi:10.1016/j.aquaculture.2008.03.020.

[37] Dulbecco R. 1952. Production of plaques in monolayer tissue cultures by single particles of an animal virus. Proc Natl Acad Sci USA 38:747–752. doi:10.1073/pnas.38.8.747. 16589172 PMC1063645

[38] Gao Y, Xiang YH, Li C, Ye J, Lu YA, Ashraf U, Liu XQ. 2021. TRIM33 promotes spring viremia of carp virus replication by degrading the antiviral protein viperin_sv1. Aquaculture 542:736837. doi:10.1016/j.aquaculture.2021.736837.

[39] Sun J, Wang J, Li L, Wu Z, Chen X, Yuan J. 2020. ROS induced by spring viraemia of carp virus activate the inflammatory response via the MAPK/AP-1 and pi3k signaling pathways. Fish Shellfish Immunol 101:216–224. doi:10.1016/j.fsi.2020.03.056. 32224280

[40] Saha S, Sadhukhan P, Sinha K, Agarwal N, Sil PC. 2016. Mangiferin attenuates oxidative stress induced renal cell damage through activation of pi3k induced Akt and Nrf-2 mediated signaling pathways. Biochem Biophys Rep 5:313–327. doi:10.1016/j.bbrep.2016.01.011. 28955838 PMC5600319

