# Supplementary material for: Mangiferin and Taurine Ameliorate MSRV Infection by Suppressing NF-κB Signaling
Source: Microbiol Spectr. 2023 May 31;11(4):e05146-22. doi: 10.1128/spectrum.05146-22 (PMC10434205; doi:10.1128/spectrum.05146-22)
Supplement: Supplemental file 1 — Figures S1 to S4. Download spectrum.05146-22-s0001.pdf, PDF file, 0.5 MB [file spectrum.05146-22-s0001.pdf]

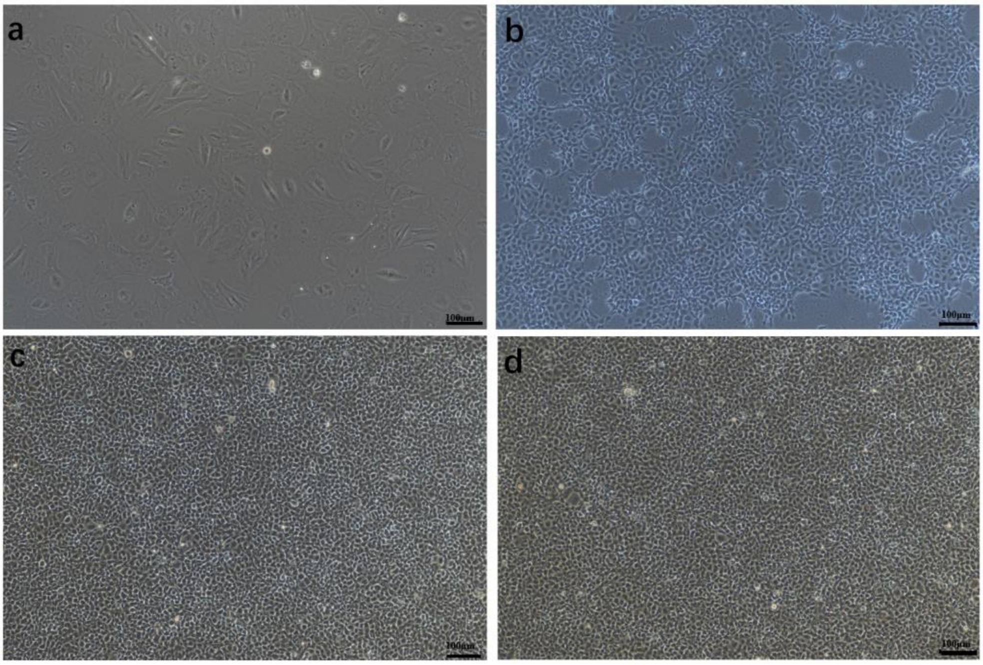

Figure S1. Morphology of the confluent *Micropterus salmoides* brain cells at passage 5 (a), passage 10 (b), passage 20 (c) and passage 60 (d) (bar=100 μm).

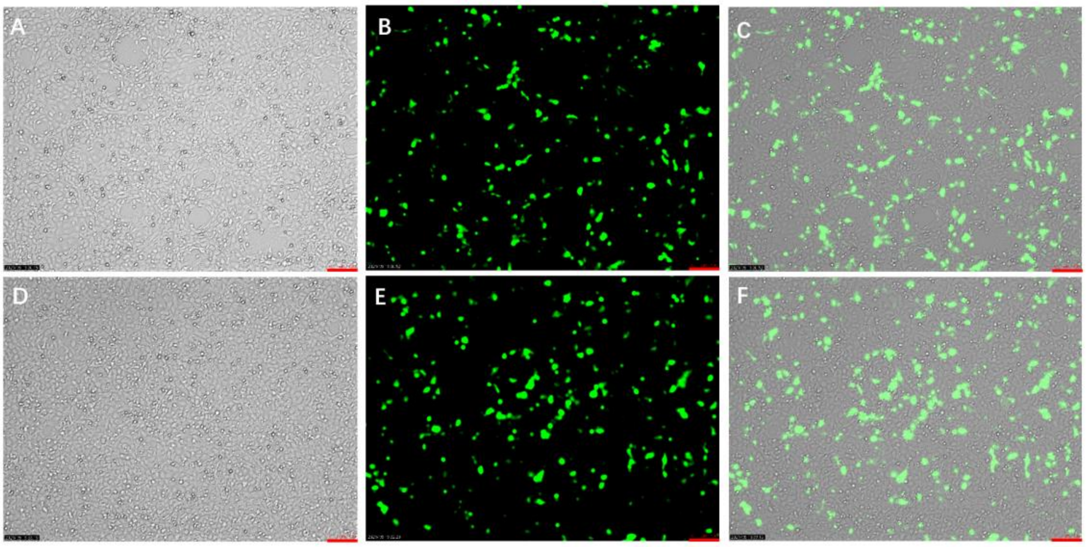

Figure S2. Expression of the GFP gene in *Micropterus salmoides* brain cells transfected with pEGFP-N1. A-C were transfected with pEGFP-N1 using Fish-trans. D-F were transfected with pEGFP-N1 using PEI reagents. A and D, White light image; B and E, fluorescence image; C and F, merged image (bar=100 μm). G, the protein (C) levels of GFP were analyzed by Western blot assays in cells, which treated with PEI and Fish-trans reagents, respectively.

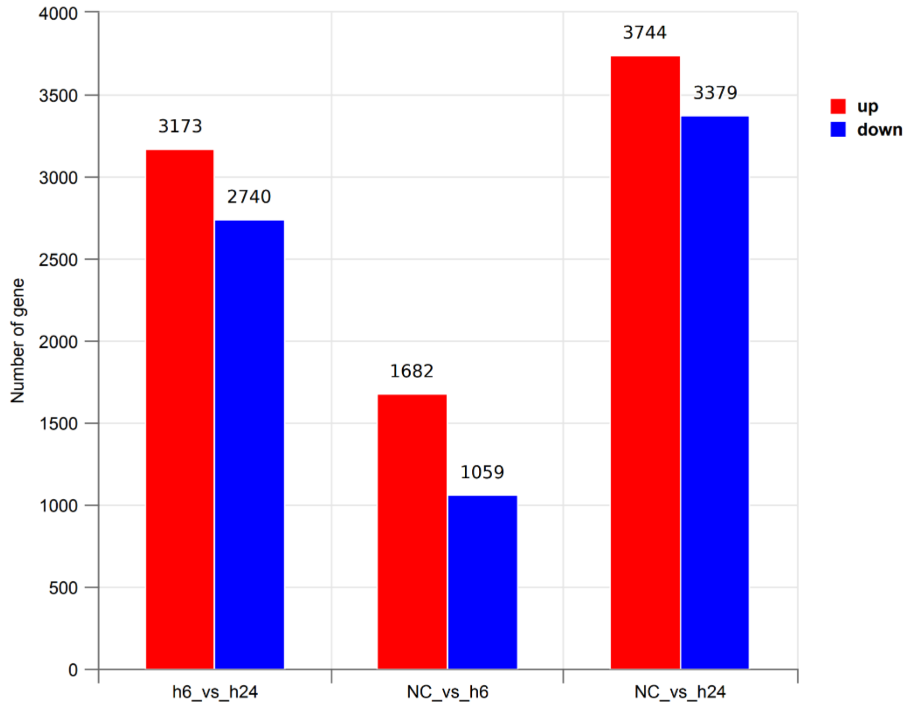

Figure S3. The number of DEGs genes involved in the pathophysiology of the *Micropterus salmoides* brain cell line (MSBr) cells response to infection of *Micropterus salmoides* rhabdovirus (MSRV).

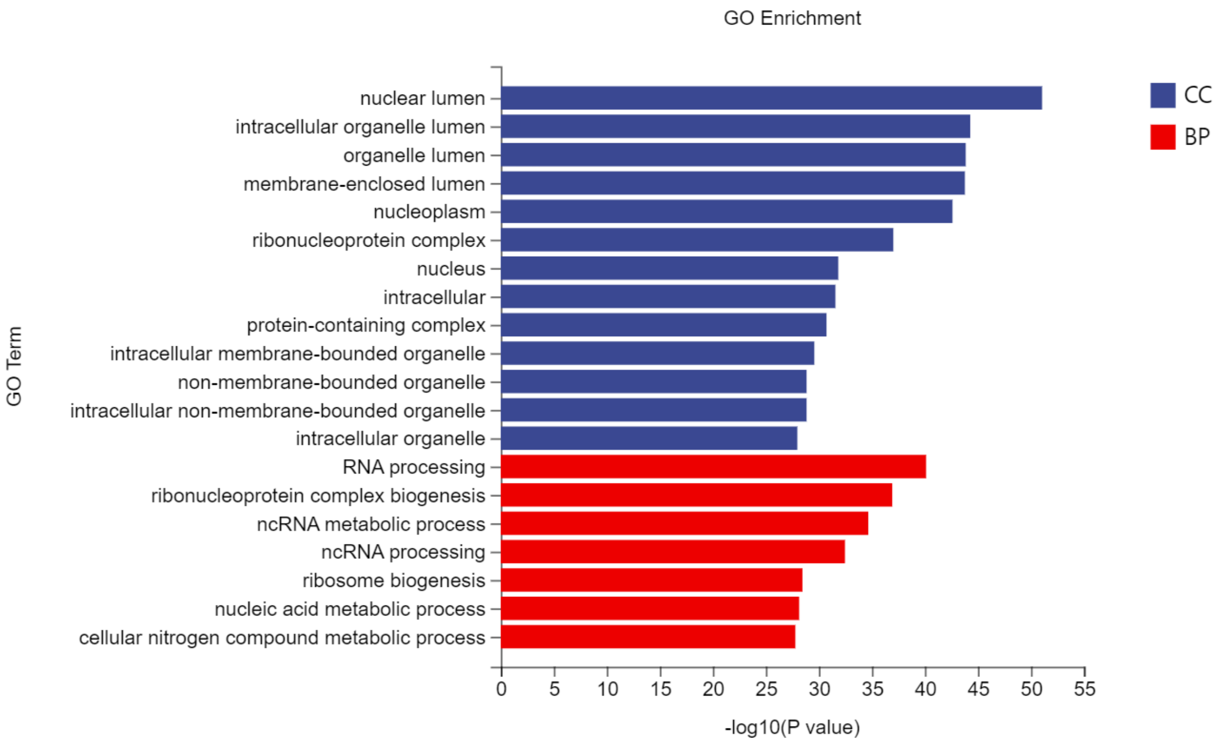

Figure S4. The top 20 level of GO terms involved in the pathophysiology of the *Micropterus salmoides* brain cell line (MSBr) cells response to infection of *Micropterus salmoides* rhabdovirus (MSRV).
